# Stress and Burnout among medical students in hainan Island, China: A cross-sectional study

**DOI:** 10.1371/journal.pone.0335682

**Published:** 2025-11-06

**Authors:** Houqian Shan, Zhaoxin Wang, Ling Yuan, Ning Chen, Maorui Yang, Di’er Cheng, Lei Sun, Zaijia Yang, Zhen Zhou, Shengfei Hou, Qingyu Wu, Hongpan Hou, Liqin Fu, Binbin Zeng

**Affiliations:** 1 School of Public Health, Hainan Medical University, Haikou, Hainan, China; 2 School of Public Health, Shanghai Jiaotong University School of Medicine, Shanghai, China; 3 UWE College of Hainan Medical University, Haikou, Hainan, China; Ajman University, UNITED ARAB EMIRATES

## Abstract

**Background:**

By 2025, the Hainan Free Trade Port is set to be fully operational, leading to a significant influx of foreign nationals into Hainan Island. This will drive a continuous increase in demand for healthcare human resources. However, many medical students currently struggle to complete their studies due to the perceived monotony of medical education and heavy academic workloads. Academic burnout has been identified as a major factor hindering students’ educational progress. Identifying factors influencing academic burnout in medical students during the early stages of their education may help reduce dropout rates. This study aims to investigate the current status of academic burnout among medical students in Hainan Province and explore associated socio-demographic factors, with the goal of providing evidence to strengthen the stability of healthcare workforce supply in Hainan, China.

**Methods:**

The final sample consisted of 551 students from a medical university in Hainan Province, China. A cross-sectional survey was conducted from June 26 to July 7, 2024. Descriptive statistics, comparative analysis, and multiple linear regression analysis were employed to investigate factors associated with stress and academic burnout among medical students at the university.

**Results:**

The mean academic stress score among respondents was 38.69 (SD = 7.88) of the participants. The mean academic burnout score was 59.96(SD = 11.03).Comparative analysis revealed no significant differences in stress levels across socio-demographic variables. However, disparities in academic burnout were observed based on:Only-child status, GPA,Maternal education level.Multiple linear regression identified predictors of academic burnout (F = 36.464, p < 0.001).Overall burnout: GPA < 2 (β = 0.120, p = 0.001), GPA 2–2.49 (β = 0.112, p = 0.002), Stress (β = 0.542, p < 0.001).The model explained 34.0% of burnout variance, with specific contributions to subdimensions:22.6% for Emotional Exhaustion,21.8% for Cynicism,29.7% for Low Academic Efficacy.

**Conclusions:**

The findings reveal concerning levels of stress and academic burnout among medical students in Hainan Province. Significant disparities in burnout were observed across socio-demographic factors, including maternal education level, only-child status, and GPA.

Stress and GPA demonstrated significant associations with academic burnout and its subdimensions emotional exhaustion, cynicism, and diminished academic efficacy. Targeted interventions to improve academic achievement among students with lower GPA may effectively alleviate burnout and subdimensions.

## Introduction

As Hainan Province approaches its 2025 milestone of becoming a fully operational free trade port, the local government has implemented streamlined market access policies for foreign medical and health equipment, alongside legal and visa facilitation measures to support international medical teams’ operations [1]. These initiatives not only provide global medical research institutions with expedited access to China’s vast healthcare market, but also drive substantial improvements in regional healthcare quality and public health infrastructure. Concurrently, they necessitate an upgraded framework for medical talent cultivation, aligning with the heightened demands of this transformative development.

The medical education process constitutes a prolonged, high-stress endeavor. The inherent monotony of medical curricula coupled with heavy academic workloads leads to significant attrition rates, resulting in substantial waste of educational resources and reduced physician workforce output. Excessive academic stress precipitates and exacerbates academic burnout – a critical determinant of learning quality in health professions education [2]. Burnout manifests through three core dimensions: emotional exhaustion, cynicism, and reduced professional efficacy [3].Medical students’ burnout demonstrates strong correlations with stress [4], with contributing factors including course load, assignments, and examination pressures [5,6]. Medical student burnout poses a significant threat not only to the students’ own mental health and professional/academic efficacy [7], but also to the healthcare workforce supply and the smooth functioning of medical systems within their regions. Significant burnout is prevalent from the outset of medical training [8]. Multi-institutional studies indicate an overall burnout prevalence rate of 37.23% among medical students [9], with at least 50% potentially meeting burnout criteria at some point during their studies [10–13]. Compared to students in other disciplines, medical students exhibit a burnout rate 14% higher [14]. Following graduation, burnout rates rise from 56% to 60% among resident physicians, showing only a slight improvement (to 51%) within the first five years of clinical practice [14]. Furthermore, medical students experience higher rates of burnout compared to their age-matched general population peers [15]. One factor associated with burnout is stress [16]. Research demonstrates that burnout, a chronic stress response, leads to dysregulation in immune, cardiovascular, neuroendocrine, and central nervous system functions [17]. Numerous studies have explored the relationship between stress and burnout [18]. Residents experience substantial stress during training, which ultimately diminishes their job satisfaction, increases burnout, and significantly negatively impacts both their mental well-being and the quality of patient care [19,20]. Importantly, research has established that medical students perceive significantly higher stress levels on average than their peers [21], and students report experiencing considerable stress starting from the very first year of medical education [22].In China, 55.16% of university students exhibit academic burnout (mild: 55.16%, severe: 3.55%, extreme: 1.28) [23]. However, no prior studies have investigated stress-burnout dynamics among Hainan medical students. This study addresses this knowledge gap by examining:Stress-burnout relationships in Hainan medical students under Free Trade Port developmentand Socio-demographic moderators of academic stress and burnout. Findings provide empirical support for designing targeted mental health interventions, crucial for sustaining healthcare workforce development in China’s biggest free trade port.

## Materials and methods

### Study design

This cross-sectional study investigated the influencing factors of academic burnout and stress among students at Hainan Medical University. The study population consisted of currently enrolled medical students from the university, with 551 participants selected through purposive sampling. Data collection was conducted via face-to-face questionnaire surveys, achieving a 0% invalid response rate. Inclusion criteria required active enrollment in medical programs with intact cognitive capacity. Exclusion criteria involved unwillingness to participate. Electronic data collection occurred from June 26 to July 7, 2024, via QR code-accessible questionnaires administered on participants’ mobile devices. Research assistants coordinated batch-wise sessions in classroom settings, projecting QR codes onto digital screens while explaining study protocols and time requirements. Written informed consent documents were distributed to all potential participants, who were required to read and sign them prior to engagement, affirming their autonomous right to determine research participation. Individuals electing non-participation could withdraw without penalty by immediately exiting the session. Participation remained strictly voluntary with no financial incentives provided for questionnaire completion. Adhering to strict confidentiality protocols, personal identifiers were systematically excluded from data collection. All participants retained unconditional withdrawal rights throughout the study duration, exercisable without providing justification.

### Instrument

All participating medical students provided socio-demographic information and completed validated academic burnout and perceived stress scales. Academic burnout was measured using the Chinese version of the Academic Burnout Scale [24]. This instrument consists of 20 items rated on a 5-point Likert scale ranging from ”completely disagree”to ”completely agree”. The total score is calculated by summing all item scores, yielding a possible range of 20–100, with higher scores indicating more severe burnout. The scale assesses three dimensions: Emotional Exhaustion, Cynicism, Low Academic Efficacy. The current study employed a Chinese version of the Academic Burnout Scale adapted from the Maslach Burnout Inventory.Numerous studies have evaluated the scale’s psychometric properties, demonstrating adequate validity for both the overall instrument and its subscales [25]. In the present study, The internal consistency reliability test yielded a total a coefficient of 0.865. The α coefficients for each dimension were as follows: Emotional Exhaustion 0.812, Cynicism 0.704, and Low Academic Efficacy 0.731. This indicates that the “Burnout Scale” has good internal consistency reliability and structural validity [24], which aligns with previous validation studies conducted in Chinese populations.

Stress levels were assessed using the 14-item scores calculated by summing all item responses (range: 14–70), categorized as low stress (14–28), moderate stress (29–56), and high stress (57–70). The scale evaluates two dimensions—perceived tension and sense of helplessness—with its validity and reliability extensively confirmed across studies [26], demonstrating high internal demonstrating high internal consistency (Cronbach’s α = 0.810) in population validations [27].

This study received ethical approval from the Ethics Committee of Hainan Medical University (Ref. ID: HYLL-2024–587). Following approval for participant recruitment, researchers approached medical students, explained the study objectives, and distributed QR codes linked to the survey questionnaires after obtaining their informed consent. Participation was entirely voluntary, with anonymity assured through non-collection of personal identifiers. All participants were guaranteed strict confidentiality of their responses in accordance with institutional data protection protocols.

### Statistical analysis

Data normality was verified using the Kolmogorov-Smirnov test, confirming a normal distribution. Reliability of the Burnout and Perceived Stress Scale-14 was assessed through Cronbach’s alpha coefficients. Analyses were conducted in SPSS Statistics 26.0, with continuous variables described as mean ± standard deviation (SD) and categorical variables as frequencies (n) and percentages (%). Total scores for academic burnout and perceived stress were calculated. Linear regression models examined associations between burnout, perceived stress, and socio-demographic variables, with statistical significance set at p < 0.05.

## Results

This study included 551 participants, with demographic characteristics showing a female predominance (64.2%) and a majority of sophomore students (62.8%), as detailed in Table 1. Participants demonstrated a mean academic stress score of 38.69 ± 7.88 and an average burnout score of 59.96 ± 11.03. While no significant stress variations were observed across socio-demographic variables. Significant differences in academic burnout emerged based on only-child status (t = −2.24,p = 0.03), GPA (t = 6.20,p < 0.001), and maternal education (t = 4.07, p = 0.02), with complete distributions presented in Table 2.

**Table 1:** Demographic characteristics of study participants.

	Characteristics	Number	Percent (%)
**Gender**			
	Male	197	35.8
	Female	354	64.2
**Background**			
	Urban	269	48.8
	Rural	282	51.2
**Only-child**			
	Yes	452	82.0
	No	99	18.0
**Student Leader**			
	Yes	189	34.3
	No	362	65.7
**Residency**			
	Hainan	243	44.1
	Mainland	308	55.9
**Ethnic**			
	Han	457	82.9
	Li	50	9.1
	Others	44	8.0
**Academic Year**			
	1^st^ Year	102	18.5
	2^nd^ Year	346	62.8
	3^rd^ Year	27	4.9
	4^th^ Year	76	13.8
**Major**			
	Clinical Medicine	139	25.2
	Preventive Medicine	58	10.5
	Laboratory Medicine	285	51.7
	Health Management	59	10.7
	others	10	1.8
**GPA**			
	<2	11	2.0
	2-2.49	43	7.8
	2.5-2.99	98	17.8
	3-3.49	211	38.3
	>3.5	188	34.1
**Paternal Education**			
	Lower Secondary	323	58.6
	Upper Secondary	110	20.0
	Tertiary	118	21.4
**Maternal Education**			
	Lower Secondary	360	65.3
	Upper Secondary	92	16.7
	Tertiary	99	18.0

**Table 2:** Analysis of burnout disparities across socio-demographic variables in medical students.

	Burnout			EE			CY			LOW AE		
	Mean	SD	t/F	Mean	SD	t/F	Mean	SD	t/F	Mean	SD	t/F
**Gender**												
Male	60.05	11.74	0.15	23.63	5.94	−0.74	18.96	4.34	1.45	17.46	4.05	−0.08
Female	59.91	10.63		23.99	5.17		18.43	3.66		17.48	3.51	
**Background**												
Urban	59.71	11.51	−0.52	23.97	5.68	0.42	18.46	4.09	−0.95	17.28	3.83	−1.18
Rural	60.20	10.57		23.77	5.24		18.77	3.75		17.66	3.59	
**Only-child**												
Yes	57.72	12.06	−2.24*	23.45	5.89	−0.83	17.42	4.08	−3.38**	16.84	3.92	−1.89
No	60.45	10.74		23.96	5.36		18.88	3.84		17.61	3.65	
**Student Leader**												
Yes	58.75	10.30	−1.86	23.33	5.29	−1.66	18.39	3.83	−1.01	17.03	3.55	−2.03*
No	60.59	11.36		24.14	5.53		18.74	3.96		17.70	3.77	
**Residency**												
Hainan	60.27	9.99	0.60	23.85	5.03	−0.07	18.70	3.6	0.41	17.73	3.48	1.43
Mainland	59.71	11.80		23.88	5.78		18.56	4.20		17.27	3.87	
**Ethnic**												
Han	59.87	11.22	0.09	23.85	5.53	0.15	18.60	3.96	0.03	17.42	3.72	0.37
Li	60.26	8.83		23.66	4.54		18.74	3.12		17.86	3.81	
Others	60.52	11.41		24.25	5.69		18.64	4.31		17.64	3.49	
**Academic Year**												
1^st^ Year	58.63	10.73	0.83	22.80	5.43	1.92	18.19	3.74	1.79	17.64	3.74	0.61
2^nd^ Year	60.19	10.91		24.00	5.36		18.72	3.89		17.47	3.66	
3^rd^ Year	61.93	13.62		23.89	6.07		19.96	4.64		18.07	3.83	
4^th^ Year	59.99	11.02		24.67	5.61		18.25	3.96		17.07	3.87	
**Major**												
Clinical Medicine	59.07	11.09	0.64	23.68	5.09	0.96	18.35	4.06	1.48	17.04	4.04	1.11
Preventive Medicine	59.16	11.27		24.40	5.56		17.72	3.88		17.03	3.78	
Laboratory Medicine	60.66	10.58		24.02	5.33		18.94	3.80		17.69	3.41	
Health Management	59.34	12.25		22.80	6.53		18.66	4.12		17.88	3.78	
others	60.50	14.67		25.20	6.55		18.00	4.03		17.30	5.77	
**GPA**												
<2	68.73	10.79	6.20	26.55	4.87	2.83	21.36	2.77	6.43	20.82	4.22	5.38
2-2.49	65.88	9.30		25.86	4.93		20.88	3.78		19.14	3.36	
2.5-2.99	60.82	11.68		24.21	5.59		18.96	4.04		17.64	3.79	
3-3.49	58.77	10.43		23.31	5.35		18.23	3.63		17.23	3.60	
>3.5	58.98	11.12		23.70	5.54		18.20	4.03		17.08	3.66	
**Paternal Education**												
Lower Secondary	60.74	10.57	2.97	23.98	5.37	0.53	18.98	3.78	4.46	17.78	3.59	3.63
Upper Secondary	59.90	11.13		24.02	5.29		18.48	4.11		17.40	3.44	
Tertiary	57.86	11.95		23.41	5.85		17.75	4.01		16.71	4.15	
**Maternal Education**												
Lower Secondary	60.60	10.75	4.07	23.98	5.37	1.67	18.89	3.88	4.06	17.74	3.60	4.60
Upper Secondary	60.50	10.66		24.36	5.24		18.64	3.69		17.50	3.43	
Tertiary	57.11	12.01		23.01	5.92		17.63	4.12		16.47	4.18	

Table notes *p < 0.05, **p < 0.01.

Table 3 displays the correlation coefficients between participants’ average stress and academic burnout, as well as the relationships between burnout dimensions and stress components. Results demonstrated a significant positive correlation between overall stress and academic burnout (r = 0.526, p < 0.01), with positive associations observed across all burnout subscales. However, statistical significance was not reached for the following relationships: the ”Low Academic Efficacy” burnout dimension with overall stress (r = 0.513), the perceived tension component of stress (r = 0.521), and the sense of helplessness stress component (r = 0.457).

**Table 3:** Correlation analysis between stress and academic burnout.

	Burnout	Emotional Exhaustion	Cynicism	Low Academic Efficacy	Press	Tension	Helplessness
**Burnout**	–						
**Emotional Exhaustion**	0.870**	–					
**Cynicism**	0.846**	0.623**	–				
**Low** **Academic Efficacy**	0.711**	0.435**	0.502**	–			
**Press**	0.526**	0.449**	0.390**	0.513	–		
**Tension**	0.541**	0.468**	0.397**	0.521	0.938**	–	
**Helplessness**	0.467**	0.394**	0.351**	0.457	0.948**	0.789**	–

Table notes ** p < 0.01.

Linear regression analysis was conducted to examine the predictive relationships between socio-demographic variables and academic burnout, assessing both the magnitude and directional effects of these predictors. The outcomes of the regression equations are comprehensively detailed in Table 4. As shown in Table 4, GPA < 2 (β = 0.120, p = 0.001), GPA 2–2.49 (β = 0.112, p = 0.002), and perceived stress (β = 0.542, p < 0.01) emerged as significant predictors of academic burnout (F = 36.464, p < 0.001) among Hainan medical students. This regression model collectively explained 34.0% of the total variance in burnout severity. Among the three dimensions of burnout, the predictive factors for the emotional exhaustion dimension were a GPA < 2 (β = 0.083, p = 0.029) and stress (β = 0.464, p < 0.001). For the cynicism dimension, significant predictors included only-child status (β = 0.109,p = 0.006), GPA < 2.0 (β = 0.100, p = 0.009), GPA 2.0–2.49 (β = 0.133, p = 0.001), and stress (β = 0.403, p < 0.001). The low academic efficacy dimension was predicted by GPA < 2.0 (β = 0.132, p < 0.001), GPA 2.0–2.49 (β = 0.178, p = 0.041), and stress (β = 0.506, p < 0.001).

**Table 4:** Analysis of burnout disparities across socio-demographic variables in medical students.

Variables	Burnout		Emotional Exhaustion		Cynicism		Low Academic Efficacy	
	β	P	β	P	β	P	β	P
**Student Leader**							0.058	0.113
**Only-child**	0.066	0.070			0.109	0.006		
**GPA(3–3.49)**								
**<2**	0.120	0.001	0.083	0.029	0.100	0.009	0.132	<0.001
**2-2.49**	0.112	0.002	0.076	0.057	0.133	0.001	0.178	0.041
**2.5-2.99**	0.066	0.085	0.061	0.138	0.066	0.114	0.031	0.429
**>3.5**	0.011	0.783	0.038	0.365	−0.005	0.897	−0.029	0.620
**Maternal** **Education(Lower Secondary)**								
**Upper Secondary**	0.031	0.400			0.044	0.32	0.013	0.756
**Tertiary**	−0.067	0.068			−0.025	0.647	−0.061	0.228
**Paternal** **Education(Lower Secondary)**								
**Upper Secondary**					−0.035	0.423	−0.024	0.559
**Tertiary**					−0.063	0.251	−0.040	0.446
**Press**	0.542	<0.001	0.464	<0.001	0.403	<0.001	0.506	<0.001
**Unadjusted R** ^**2**^		0.350		0.233		0.233		0.310
**Adjusted R** ^**2**^		0.340		0.226		0.218		0.297
**F**		36.464		33.042		16.361		24.227

The regression model revealed that GPA < 2.0, GPA between 2.0–2.49, and stress significantly predicted academic burnout, collectively accounting for 34.0% of the variance in overall burnout. Regarding emotional exhaustion, GPA < 2.0 and stress emerged as predictors of cynicism, explaining 22.6% of the variance in emotional exhaustion. For the cynicism dimension, only-child status (β = 0.09), GPA < 2.0, GPA 2.0–2.49, and stress jointly predicted behavioral disengagement, contributing to21.8% of the variance in cynicism. In terms of low academic efficacy, GPA < 2.0, GPA 2.0–2.49, and stress were all significant predictors, explaining 29.7% of the variance in this dimension (Table 4).

## Discussion

This study investigated the relationship between stress and academic burnout among medical students. The results showed that the mean stress score of participants was 38.69 ± 7.88, with 34.84% (n = 192) scoring 43−56 indicating high stress levels, and 0.54% (n = 3) scoring 57 demonstrating clinically significant stress. These findings indicate that the surveyed medical students exhibited moderate stress levels, which aligns with Sarah Salih’s research [28] reporting comparable stress profiles in medical students. However, divergent findings exist in other studies where stress prevalence reached 66.8% [29]. This discrepancy may be attributed to the COVID-19 pandemic context, during which medical students faced heightened stress due to pandemic-induced mortality rates and frontline healthcare challenges.

This study revealed medical students’ mean academic burnout score of 59.96 ± 11.03 points. Using the sample median (60points) as the cutoff, 60.66% of participants exceeded this threshold indicating significant burnout. These results suggest elevated academic burnout levels among medical students in Hainan.The literature indicates that many medical students are at risk of burnout or are currently suffering from it [30–32]. However, some literature shows that the overall burnout level among medical students is only 14% [33]. Zaidi reported that the incidence of burnout among medical students reached 52.5% [34]. In a national survey in Israel, the incidence of academic burnout among medical students was 50.6% [35]. Another survey conducted in Romania reported a burnout prevalence rate of 47.8% among medical students [36]. Medical students face numerous stress-related challenges during their studies. These challenges include heavy academic workloads, examinations, internships in hospitals, and night-shift internships. To adjust the mental health status of medical students and alleviate burnout, it is necessary to make adjustments and plans for the medical curriculum. Medical students are usually in a vulnerable stage of life (aged 18–24), during which they are more prone to mental health problems [37].

Also, the study revealed significant variations in academic burnout levels across different GPA groups, with lower-academic performing students demonstrating higher burnout scores, necessitating institutional focus on academically at-risk populations—a pattern consistent with Calcides [38] and Puranitee [39], where academic underachievement and examination failures were identified as burnout catalysts [12]. Contrary to Thun-Hohenstein and Gil-Calder´on’s reports [40,41], no gender- or grade-level disparities in burnout were observed. Methodologically, convenience sampling may introduce selection bias in this research. The results of this study show that different educational levels of parents can affect students’ burnout. The higher the educational level of the mother, the lower the students’ scores in the burnout, dimensions of Cynicism and Low Academic Efficacy. In the existing literature investigation, obtaining support from the family is also a protective factor for students to alleviate burnout [42], and feeling unable to obtain support from the family can also be a protective factor for students to alleviate burnout [43].

This reseach demonstrated a positive correlation between stress and academic burnout scores, specifically indicating that increased stress levels and increased burnout at the same time—a pattern consistent with prior observations in other populations [44,45], suggesting that when doctors are in a state of high professional burnout, their sense of stress is also at a high level. When stress increases, burnout also reaches a high level [46]. Medical education’s unique demands, extending beyond theoretical memorization to intensive basic medical experimentation, significantly amplify academic pressures. In a study investigating the role of stress in burnout, Burnout during rural placement may be a consequence of stress prior to a medical school the placement [47], and the transition to the clinical internship stage is highly stressful for medical students [48].

The regression results are same with the research results of seo [49] and Popa-Velea [45]. Stress can predict academic burnout, similarly, a GPA < 2 and GPA from 2–2.49 can predict academic burnout. The research by Puranitee shows a negative correlation between GPA and academic burnout [39]. Aljadani’s regression study indicates that a higher GPA leads to a lower burnout score [50], and academic performance is also a protective factor for burnout symptoms [51]. To explain this finding, it can be considered that stress and academic performance are crucial to the emergence of burnout symptoms. A lower sense of stress and higher academic performance provide a basis for the reduction of burnout. If the sense of stress among medical students is reduced, their sense of burnout in their studies will be alleviated, and the improvement of academic performance will also affect sense of burnout. When studying the relationship between burnout and academic performance, Thew pointed out that not only grades but also excessive examinations can cause learning burnout [52]. Therefore, helping medical students reduce stress and improve their academic performance could help them avoid academic burnout.

The study demonstrated relationships across burnout dimensions: GPA < 2 and stress significantly predicted emotional exhaustion; only-child status, GPA < 2, GPA 2–2.49, and stress influenced cynicism; while GPA < 2, GPA 2–2.49, and stress predicted low academic efficacy. These findings align with Obregon’s identification of curriculum misalignment as causal to emotional exhaustion/cynicism [53], and Seo’s confirmation of educational/relational stressors impacting all burnout dimensions [50]. Notably, Popa-Velea identified stress as the strongest predictor of exhaustion/low academic efficacy, with perceived social support deficits (particularly among females) driving cynicism [51]. In the research university, Institutional policy changes—shifting from a 40% written exam/60% regular performance pass standard (pre-March 2024) to mandatory 60% written exam thresholds under April’s medical education reform—exacerbated academic stress and indirectly intensified burnout. Contradictory evidence suggests academic performance may only correlate with burnout, as higher GPA students (3.51–4.00) showed lower total burnout scores [53].

Medical students should be cognizant that sustained academic stress and suboptimal academic performance (GPA < 2) substantially increase burnout risks, with chronic underachievement potentially triggering psychological distress through repeated academic setbacks—necessitating systematic interventions targeting burnout etiology for successful educational outcomes. Medical institutions must implement curricular reforms including streamlined curricula, reduced assignment loads, and enhanced extracurricular engagement, while recruiting PhD-qualified faculty to improve instructional quality. Concurrently, stress mitigation requires infrastructural enhancements: provision of sufficient study spaces with air conditioning and drinking water, need-based scholarships for economically disadvantaged students, and institutionalization of mindfulness-based stress reduction programs through campus psychological services. These evidence-based strategies—shown to reduce burnout prevalence—not only foster mental health resilience but also improve licensure exam pass rates, ultimately supporting healthcare workforce development for the Hainan Free Trade Port initiative through increased graduate competency retention.

This investigation has several notable limitations, primarily its single-center design at one medical university which restricts generalizability, compounded by convenience sampling methodology that may introduce selection and sampling biases—thus caution is warranted in interpreting the findings given the non-randomized participant selection and absence of multi-institutional representation. Additional constraints include unmeasured confounders such as personality traits, pre-existing mental health conditions, and medication histories that might influence stress-burnout dynamics.

Future longitudinal multicenter studies should incorporate comprehensive psychosocial assessments to identify novel mediators in medical students’ stress-burnout pathways while controlling for these omitted variables.

## Conclusion

This study revealed significant disparities in medical students’ academic burnout across socio-demographic variables, with only-child status, GPA, and maternal education level demonstrating differential impacts, while stress exhibited consistent correlations across all dimensions. Specifically, emotional exhaustion showed GPA-stratified differences (GPA < 2, GPA 2–2.49) and stress association. Cynicism varied by only-child status, GPA thresholds (GPA < 2, 2–2.49), and parental education correlating with stress. Reduced academic efficacy displayed class-leadership, GPA (GPA < 2, 2–2.49), and dual-parent education effects (maternal, paternal), maintaining stress correlation. The predictive capacity of stress and GPA on medical students’ burnout necessitates institutional reforms in curricular structures and pedagogical approaches to mitigate stressors and enhance academic performance. Notably, only-child status emerged as a significant predictor in the cynicism dimension compelling medical educators to integrate these socio-academic determinants when redesigning training programs for burnout prevention. Therefore, medical students should receive support from administrators to reduce stress and improve academic performance in order to reduce academic burnout.
